# Sepsis-related myocardial injury: the role of bacterial pathogens and toxins—a scoping review protocol

**DOI:** 10.1136/bmjopen-2025-102485

**Published:** 2025-10-07

**Authors:** Samantha Lörstad, Jacob Widaeus, Patrik Gille-Johnson, Jonas Persson

**Affiliations:** 1 Department of Clinical Sciences, Danderyd University Hospital, Division of Internal Medicine, Karolinska Institute, Stockholm, Sweden; 2 Division of Infectious Diseases, Danderyd University Hospital, Stockholm, Sweden; 3 Department of Clinical Sciences, Danderyd University Hospital, Division of Cardiovascular Medicine, Karolinska Institute, Stockholm, Sweden

**Keywords:** Sepsis, Cardiomyopathy, Adult intensive & critical care, Cardiology, Shock, Septic, BACTERIOLOGY

## Abstract

**Abstract:**

**Introduction:**

Sepsis-related myocardial injury is common in sepsis patients and has been repeatedly associated with poor patient outcomes. While experimental animal studies suggest that direct and indirect myocardial damage occurs via a wide range of inflammatory mediators, including bacterial toxins, the extent of bacterial-driven myocardial injury in human sepsis remains unclear. This scoping review aims to map existing evidence, identify knowledge gaps and guide future research priorities.

**Methods and analysis:**

This scoping review will follow the Joanna Briggs Institute methodology for scoping reviews. The review is anticipated to start on 12 December 2024 and be completed by 31 October 2025. A comprehensive search will be conducted in MEDLINE (PubMed), Web of Science and EMBASE. Two independent reviewers will screen titles and abstracts, followed by a full-text review of potentially eligible studies. Studies focusing on the role of bacteria and/or their toxins in myocardial injury in adult patients with sepsis will be included. Data will be independently extracted using predefined forms, and findings will be reported in accordance with the Preferred Reporting Items for Systematic Reviews and Meta-analysis extension for Scoping Reviews guidelines.

**Ethics and dissemination:**

Ethical approval is not required for this scoping review. On completion, findings will be submitted for publication in a peer-reviewed medical journal and presented at scientific conferences. The results will help identify and analyse knowledge gaps, providing valuable insights to inform future research in patients with sepsis.

**Registration details:**

Registered on Open Science Framework 3 March 2025.

**STRENGTHS AND LIMITATIONS OF THIS STUDY**

- This review will follow a systematic approach, with data extraction guided by the Joanna Briggs Institute methodology manual.
- The protocol has been developed with input from a multidisciplinary team of cardiology, infectious diseases and critical care physicians, as well as experienced medical librarians.
- The search strategy has been designed to be broad and inclusive, covering multiple databases and both historical and contemporary literature.
- A potential limitation is the heterogeneity across studies in trial design, sampled cardiac biomarkers and assessment of myocardial injury and dysfunction.

## Introduction

 Sepsis is a life-threatening condition characterised by a dysregulated systemic inflammatory response to infection, often leading to multiorgan dysfunction due to hyperinflammation-induced microcirculatory changes.^1 2^ Among sepsis complications, sepsis-related myocardial injury has gained increasing attention due to its association with poorer patient outcomes, including post-sepsis cardiac events and worse survival in survivors.^3^,^5^

Sepsis-induced myocardial injury is thought to result from both direct and indirect damage to the myocardium, driven by a wide range of inflammatory mediators, including bacteria, bacterial products and, in particular, their toxins.^6^,^9^ Recent microbiological advances in experimental animal models have established that invading pathogens are recognised by pattern recognition receptors, such as Toll-Like Receptors (TLRs) expressed on host immune cells. These receptors detect microbial molecular structures known as pathogen-associated molecular patterns (PAMPs), initiating intracellular signalling cascades that activate anti-pathogen immune responses.^10^,^12^ Key PAMPS include: (1) Lipopolysaccharide (LPS), a Gram-negative bacterial endotoxin, (2) Lipoteichoic acid, a Gram-positive bacterial cell wall component and (3) Flagellin, the structural protein of bacterial flagella.^10^

In experimental sepsis models, multiple TLRs have also been shown to be expressed on cardiomyocytes. Activation of TLR2, TLR4 and TLR5 has been linked to reduced cardiomyocyte contractility and nuclear factor kappa B mediated inflammation.^13^ Specifically, TLR4 has been implicated in both myocardial ischaemia and sepsis-induced myocardial dysfunction, TLR2 in contractility loss during *Staphylococcus aureus* sepsis, and TLR5 in innate immune activation in rat cardiomyocytes.^12^,^15^

Animal models have provided important insights into intracellular signalling pathways involved in sepsis-related myocardial injury. In addition to TLR activation, calcium dysregulation, mitochondrial dysfunction, oxidative stress, maladaptive activation of cardiomyocyte apoptosis and dysregulated autophagy have all been implicated in myocardial impairment.^16^,^18^ Over the past five decades, advances in biomarkers of myocardial injury have refined diagnostic approaches. Early markers such as aspartate aminotransferase and lactate dehydrogenase lacked specificity, while creatine kinase-MB (CK-MB) improved sensitivity but remained limited by skeletal muscle expression.^19^ The introduction of cardiac-specific troponins (cTnI, cTnT) revolutionised acute myocardial infarction diagnosis, offering superior specificity and sensitivity.^20^

In sepsis, elevated troponin levels are associated with worse prognosis, though the mechanisms underlying their release remain unclear.^5 21 22^ The *Fourth Universal Definition of Myocardial Infarction (2018*) acknowledges that ‘patients may have elevated cTn values and marked decreases in ejection fraction due to sepsis caused by endotoxin, with myocardial function recovering completely once the sepsis is treated’.^23^ Despite their clinical utility, elevated cardiac biomarkers alone do not confirm myocardial injury or ventricular dysfunction. Histopathological evidence remains the definitive proof of myocardial injury, but myocardial tissue is rarely available in clinical practice, making imaging indispensable. Coronary angiography, echocardiography and MRI are therefore essential for differentiating the underlying cause, assessing severity and guiding management.^23^

The first echocardiographic evidence of acute myocardial depression in sepsis emerged in the 1980s, describing reversible left ventricular dysfunction.^24^ Around the same time, a canine model study demonstrated left ventricular abnormalities on echocardiography, irrespective of whether the pathogen was Gram-negative (*Escherichia coli*) or Gram-positive (*Staphylococcus aureus*).^25^ Further research by the same group also established a dose-dependent relationship between *Escherichia coli* bacterial load and reduced myocardial compliance and contractility.^26^ In contrast, a human study in septic shock found that early myocardial depression was significantly more common in *Neisseria meningitidis* infections than in other Gram-negative pathogens.^27^ More recent human studies show a more complex biventricular profile on echocardiography of sepsis-related myocardial dysfunction, with uncertain long-term implications.^28^,^31^

Studying myocardial injury at the cellular level presents considerable technical challenges, necessitating the use of experimental animal models. Key approaches include histopathological analysis of myocardial tissue and light and electron microscopy of cardiac muscle fibres and cardiomyocytes. Most models replicate sepsis-induced immune responses by administering LPS, lipoteichoic acid or live pathogens intravenously.^32^,^34^ However, both LPS and lipoteichoic acid appear to primarily trigger immune responses rather than exert direct cytotoxic effects.^35 36^ Furthermore, human sepsis typically involves only minimal bacterial or endotoxin translocation, raising concerns about how accurately these models replicate clinical sepsis.^37^

Among the available experimental models, the caecal ligation and puncture (CLP) model, which mimics faecal peritonitis, has become widely used in sepsis research.^35 37^ Compared with LPS or single-pathogen models, CLP better represents the polymicrobial nature of human sepsis, yet it remains criticised for not fully capturing the clinical heterogeneity and complexity of the condition.^35 37^

Several toxin-producing bacteria have been implicated in direct cardiomyocyte injury in experimental sepsis models, including *Corynebacterium diphtheriae* (rat/guinea pig),^38^ *Streptococcus pyogenes* (murine),^39^ *Streptococcus pneumoniae* (murine),^40^ *Staphylococcus aureus* (rat),^41^ *Vibrio cholerae* (rat)^42^ and *Escherichia coli* (guinea pig).^43^ Together, these findings from experimental models suggest that bacterial toxins may directly injure cardiomyocytes, but their translational relevance remains uncertain, reinforcing the need to synthesise the human evidence base systematically.

This scoping review aims to summarise current literature on the direct toxicity of bacteria and bacterial toxins on the human myocardium and their role in sepsis-related myocardial injury. While sepsis may result from diverse pathogens, including viruses, parasites and fungi, these aetiologies fall outside the scope of the present review. Two preliminary searches of MEDLINE (PubMed), the Cochrane Database of Systematic Reviews and Joanna Briggs Institute (JBI) Evidence Synthesis (conducted in November 2024 and February 2025) found no existing or ongoing systematic or scoping reviews on this topic. Furthermore, targeted searches with narrower research questions yielded only a few human studies with diverse designs and methodologies. Given this, a scoping review was deemed the most appropriate approach at this stage to map existing studies, summarise the evidence and identify knowledge gaps, rather than applying the rigorous methodology required for a systematic review.

## Methods and analysis

The methods for this review were developed in accordance with the JBI scoping review methodology.^44^ The key components of the review process are detailed below. This scoping review was registered with Open Science Framework (https://osf.io/) on 3 March 2025. The review is anticipated to start on 12 December 2024 and be completed by 31 October 2025.

### Research question

This scoping review aims to explore the role of bacteria and bacterial toxins in myocardial injury in sepsis patients. Specifically, it will identify which bacterial species and toxins have been associated with myocardial injury and examine the underlying cellular mechanisms. Additionally, the review will map the methods used to detect bacteria-induced myocardial injury, including laboratory tests, histopathological analyses and cardiac imaging techniques. The strength of the evidence supporting any identified associations will be assessed by evaluating study designs and their clinical relevance to sepsis populations. Finally, the review will highlight unanswered questions in this field, identifying key knowledge gaps that require further research.

### Inclusion criteria

The inclusion criteria were formulated based on the ‘Population-Concept-Context’ framework recommended by the JBI.^44^ This scoping review will include studies on adult human subjects (aged ≥18 years) diagnosed with sepsis or experimentally induced bacteraemia or endotoxaemia. Eligible studies must investigate the effects and mechanisms by which bacteria and/or bacterial toxins contribute to myocardial injury and incorporate an assessment of cardiomyocyte or ventricular function. Studies from all countries and time periods will be considered.

The *Fourth Universal Definition of Myocardial Infarction (2018*) defines myocardial injury strictly on the basis of cardiac troponin elevation.^23^ However, in the context of sepsis, both historical and contemporary studies have used a broader range of biomarkers, including CK-MB, myoglobin and natriuretic peptides (BNP, NT-proBNP), to indicate myocardial involvement. To ensure comprehensive coverage of the available evidence, this review will include studies reporting myocardial injury according to the definitions applied by the original authors, even when based on these alternative biomarkers. For consistency and transparency, the biomarker type and threshold reported will be recorded during data extraction and distinguished in the synthesis.

The review will include clinical trials, cohort studies (including case-control and observational cohorts), case reports and laboratory studies that evaluate myocardial injury through serum cardiac biomarker release and/or histopathological cardiomyocyte changes. Additionally, studies must provide evidence of cardiomyocyte injury, coronary microvascular dysfunction or ventricular dysfunction, assessed by histopathology, electrocardiography or imaging techniques such as coronary angiography, echocardiography or MRI, including, where available, measurements of coronary flow or myocardial perfusion.

### Exclusion criteria

Studies will be excluded if they are animal studies or if they focus solely on bacteria-induced or bacterial product-induced inflammatory cascades rather than direct myocardial injury or toxicity. Paediatric populations (<18 years) will be excluded because sepsis in children differs from adults in epidemiology, pathophysiology, biomarker thresholds and echocardiographic criteria, making direct comparison inappropriate. Research on bacterial endocarditis, viral myocarditis, aortitis, cardiac abscesses or mycotic aneurysms will also be excluded. Furthermore, studies primarily involving patients undergoing pharmacological interventions, extracorporeal therapies (eg, haemoperfusion or extracorporeal membrane oxygenation), transplantation, open-heart surgery or burn-related sepsis will not be considered.

### Search strategy

A comprehensive literature search will be conducted using MEDLINE (PubMed), Web of Science and Embase. The search will be guided by the major concepts: ‘bacteria/bacterial toxins’, ‘myocardial injury’ and ‘sepsis’. The full search strategy is detailed in online supplemental appendix 1. Highly specific imaging terms (eg, cardiac output, left ventricular ejection fraction) were not included in the search string, as test searches showed that their use markedly reduced sensitivity and excluded potentially relevant studies. Following the advice of a medical librarian, we therefore applied broader MeSH terms and text words to ensure comprehensive coverage of the literature.

With the assistance of an experienced health science librarian, MeSH terms (for MEDLINE) were identified, followed by the extraction of relevant keywords and entry terms across MEDLINE, Web of Science and Embase. To ensure reproducibility, a second health science librarian validated the search strategy.

No restrictions on time period or language will be applied; however, studies will be manually limited to the English language during the selection process. All reference management will be performed using the online platform Covidence (2025 Covidence, Melbourne, Australia).

To identify additional relevant sources, the reference lists of included studies and relevant reviews will be screened. If necessary, authors of primary studies will be contacted for further information. To ensure the inclusion of the most recent literature, searches will be updated prior to publication.

### Evidence selection

Study selection will be conducted systematically using Covidence, following the predefined inclusion and exclusion criteria. After the removal of duplicates, two reviewers (SL and JW) will independently screen all articles.

The initial screening will be based on titles and abstracts. Disagreements at this stage will be resolved through discussion between the two reviewers. If uncertainty persists, a third reviewer (JP) will be consulted for a final decision. Final inclusion will be determined through full-text screening, with disagreements resolved in the same manner as the title/abstract stage.

The initial database searches were conducted in December 2024, identifying 14 182 studies after deduplication. Full-text screening is currently ongoing.

### Data extraction

Data extraction will be conducted independently by two reviewers (SL and JW). Key information from the selected articles will be extracted using a predefined data extraction spreadsheet, designed specifically for this review and provided in the online supplemental appendix 2. This form, adapted from a JBI template, will be refined as necessary throughout the review process.^44^

### Analysis of the evidence and presentation of the results

The findings of this review will be presented through narrative synthesis, following the Preferred Reporting Items for Systematic Reviews and Meta-analysis Protocols Extension for Scoping Reviews (PRISMA-ScR).^45^ A tabular overview of the reviewed literature will be provided, complemented by visual summaries and descriptive statistics. If relevant, evidence and/or gap maps may be included to highlight key findings and research gaps. Although quality assessment is not an obligatory component of scoping reviews, we plan to critically appraise the evidence using validated tools such as the Cochrane Risk of Bias Tool (RoB2) for randomised trials and the Newcastle-Ottawa scale quality assessment scale for cohort studies. This review aims to serve as a foundation for identifying important research questions that can guide future primary studies and systematic reviews.

### Patient and public involvement

This research will be done without patient and public involvement.

### Ethics and dissemination

As this scoping review involves the analysis of publicly available data, ethical approval is not required. The findings will be disseminated through a peer-reviewed publication and presented at conferences to reach a broader audience.

## Supplementary material

10.1136/bmjopen-2025-102485 online supplemental file 1

10.1136/bmjopen-2025-102485 online supplemental file 2

## Data Availability

All data relevant to the study are included in the article or uploaded as supplementary information.
