# Supplementary material for: Sepsis-related myocardial injury: the role of bacterial pathogens and toxins—a scoping review protocol
Source: BMJ Open. 2025 Oct 7;15(10):e102485. doi: 10.1136/bmjopen-2025-102485 (PMC12506174; doi:10.1136/bmjopen-2025-102485)
Supplement: online supplemental file 2 [file bmjopen-15-10-s002.docx]

| **Author, Year** | **Country** | **Study Design** | **Setting** | **Study Aim** | **Sample Size (n=)** | **Age (years)** | **Sex** | **Sepsis criteria** | **Pathogen type** | **Specific pathogen(s)** |
| --- | --- | --- | --- | --- | --- | --- | --- | --- | --- | --- |
|  |  |  |  |  |  |  |  |  |  |  |

**Appendix 2**

**(i) Data extraction table**

| **Serum cardiac biomarkers** | **Functional myocardial assessment** | **Histopathology or molecular markers** | **Key findings** | **Reported associations** | **Conclusions** | **Clinical implications** |
| --- | --- | --- | --- | --- | --- | --- |
|  |  |  |  |  |  |  |
