# Supplementary material for: Sepsis-related myocardial injury: the role of bacterial pathogens and toxins—a scoping review protocol
Source: BMJ Open. 2025 Oct 7;15(10):e102485. doi: 10.1136/bmjopen-2025-102485 (PMC12506174; doi:10.1136/bmjopen-2025-102485)
Supplement: Supplementary file 3 [file bmjopen-2025-102485.reviewer_comments.pdf]

PEER REVIEW HISTORY

BMJ Open publishes all reviews undertaken for accepted manuscripts. Reviewers are asked to complete a checklist review form and are provided with free text boxes to elaborate on their assessment. These free text comments are reproduced below.

ARTICLE DETAILS

Title (Provisional)

Sepsis-related myocardial injury: the role of bacterial pathogens and toxins - a scoping review protocol

Authors

Lörstad, Samantha; Widaeus, Jacob; Gille-Johnson, Patrik; Persson, Jonas

---

VERSION 1 - REVIEW

**Reviewer**	**1**
**Name**	**Doyen, Denis**
**Affiliation**	**University Hospital Centre Nice, Médecine Intensive Réanimation**
**Date**	**05-Jul-2025**
**COI**	**None**

The authors present a review protocol aimed at describing the role of bacteria and their toxins in sepsis-related myocardial injury and dysfunction.

The topic is original and addresses an important clinical question. This objective is particularly challenging, as sepsis-related myocardial injury poorly described with various definitions and descriptions in the literature (doi:10.1097/CCM.0000000000002851, doi:10.1186/s13054-018-2043-8).

I have several comments:

- the nomenclature “sepsis-related myocardial injury and dysfunction” is confusing and questionable. I would suggest using the simpler term “sepsis-related myocardial injury,” which inherently implies the possible presence of cardiac dysfunction. Various other terms are used in the literature, such as “septic cardiomyopathy” or “sepsis-induced myocardial dysfunction” (doi:10.1097/CCM.0000000000002851, doi:10.1186/s13054-018-2043-8). The authors might consider adopting one of these terms for better consistency with existing literature.

- The authors should provide a clear definition of "sepsis-related myocardial injury." For example, does it refer to troponin elevation with or without abnormalities detected by imaging techniques? Or is the presence of any of these abnormalities sufficient ?

Electrocardiogram is a simple and widely used examination, and electrocardiographic abnormalities are commonly employed to define various cardiomyopathies (doi:10.1161/CIR.0000000000001062). The authors should explain why they chose not to include electrocardiographic abnormalities in their screening criteria

- In the Introduction, some referenced studies (e.g., references 10 to 13) were conducted in mice rather than humans. The authors should specify the experimental model used for each affirmation

- Why did the authors limit inclusion to patients over 18 years of age? As they noted, a retrospective analysis does not raise ethical concerns regarding pediatric populations

- Patients with a known history of cardiomyopathy should be excluded from the analysis to ensure that any abnormal cardiologic findings are specific to sepsis-related myocardial injury and not due to pre-existing cardiac conditions

- Regarding the search strategy, terms related to cardiac abnormalities are missing. Other studies have reported the use of various imaging techniques in the assessment of sepsis-related myocardial injury (doi:10.1097/CCM.0000000000002851, doi:10.1186/s13054-018-2043-8), including echocardiography, magnetic resonance imaging, left ventricular ejection fraction, Tei index, tricuspid annular plane systolic excursion, tricuspid S' peak systolic velocity, and cardiac output

- Sepsis can be caused not only by bacteria but also by other microorganisms such as viruses, parasites, and fungi. The authors should justify why their analysis focuses exclusively on bacterial causes.

**Reviewer**	**2**
**Name**	**Rozec, Bertrand**
**Affiliation**	**University hospital of Nantes**
**Date**	**04-Aug-2025**
**COI**	**None**

Here, the authors propose their methodology for carrying out a scoping review of the cardiac effects of bacterial toxins (direct effect) during septic shock in humans

Although the subject itself is particularly interesting and the inclusion and exclusion criteria and the review methodology are very well described in accordance with rigorous standards and approaches, I fail to understand the point of publishing the protocol for this review. This is a personal opinion that only I can express. Once again, I find the subject fascinating and

one that could have a major impact on developing future research projects: targeted and personalised therapy, biomarker research, personalised approach to the host-pathogen relationship, etc. So yes, providing information about such a research project is interesting, but the methodology is less exciting, especially in the age of AI.

VERSION 1 - AUTHOR RESPONSE

Reviewer: 1

1) the nomenclature "sepsis-related myocardial injury and dysfunction" is confusing and questionable. I would suggest using the simpler term "sepsis-related myocardial injury," which inherently implies the possible presence of cardiac dysfunction. Various other terms are used in the literature, such as "septic cardiomyopathy" or "sepsis-induced myocardial dysfunction" (doi:10.1097/CCM.0000000000002851, doi:10.1186/s13054-018-2043-8). The authors might consider adopting one of these terms for better consistency with existing literature.

We thank the reviewer for this valuable comment. We agree that there is considerable heterogeneity of terminology in this field, with many terms used inconsistently and without clear definitions. Our deliberate focus on patients with both biomarker evidence of myocardial injury and imaging-assessed dysfunction is directly supported by the Fourth Universal Definition of MI (2018), which states: *"Other patients may have elevated cTn values and marked decreases in ejection fraction due to sepsis caused by endotoxin, with myocardial function recovering completely once the sepsis is treated."* (PMID: 30153967). Despite their clinical utility, elevated cardiac biomarkers alone cannot confirm myocardial injury or ventricular dysfunction. Histopathological examination remains the definitive proof of myocardial injury, but myocardial tissue is rarely available in clinical practice, making imaging indispensable. Coronary angiography and echocardiography are therefore essential for differentiating the underlying cause, assessing severity, and guiding management. Our study was specifically designed to capture this dual presentation, which we believe represents a more clinically and biologically meaningful entity than either injury (studies limited to cardiac biomarkers) or dysfunction alone (studies limited to echocardiographic results). To better-define our objective, reasoning and nomenclaturen we have made the following revisions to the manuscript:

- The title has been altered and reads: "Sepsis-related myocardial injury: the role of bacterial pathogens and toxins - a scoping review protocol" (page 1, lines 2-3)
- The phrase "sepsis-related myocardial injury and dysfunction" has been reduced to "sepsis-related myocardial injury" throughout the manuscript.
- The following change has been made to clarify the nomenclature: "This scoping review aims to summarize current literature on the direct toxicity of bacteria and bacterial toxins on the human myocardium and <u>their role in sepsis-related myocardial injury"</u> (page 6, lines 13-15).

2) The authors should provide a clear definition of "sepsis-related myocardial injury." For example, does it refer to troponin elevation with or without abnormalities detected by imaging techniques? Or is the presence of any of these abnormalities sufficient ?

- We thank the reviewer for raising this important point. We acknowledge that the *Fourth Universal Definition of MI (2018)* defines myocardial injury strictly on the basis of cardiac troponin elevation. However, in the context of sepsis, both historical and contemporary studies have employed a wider range of biomarkers, including CK-MB, myoglobin, and natriuretic peptides (BNP, NT-proBNP). To ensure comprehensive coverage of the available evidence, our review will therefore include studies that defined myocardial injury according to these biomarkers as reported by the original authors. For consistency and transparency, we will record and distinguish biomarker type during data extraction and interpretation. To clarify this important point we have inserted the following paragraph in the "Methods and Analysis" section:

  "The *Fourth Universal Definition of Myocardial Infarction (2018)* defines myocardial injury strictly on the basis of cardiac troponin elevation. However, in the context of sepsis, both historical and contemporary studies have used a broader range of biomarkers, including CK-MB, myoglobin, and natriuretic peptides (BNP, NT-proBNP), to indicate myocardial involvement. To ensure comprehensive coverage of the available evidence, this review will include studies reporting myocardial injury according to the definitions applied by the original authors, even when based on these alternative biomarkers. For consistency and transparency, the biomarker type and threshold reported will be recorded during data extraction and distinguished in the synthesis." (Page7, lines 21-29)

- We already state in the original manuscript that: "Despite their clinical utility, elevated cardiac biomarkers alone do not confirm myocardial injury or ventricular dysfunction. Assessment of coronary anatomy with coronary angiography and echocardiography are essential for differentiating the underlying cause, assessing severity, and guiding management" (Page 4, line 29 – Page 5, line 2). To further clarify, the paragraph has been essentially re-written and reads:
  "In sepsis, elevated troponin levels are associated with worse prognosis, though the mechanisms underlying their release remain unclear. The *Fourth Universal Definition of Myocardial Infarction (2018)* acknowledges that "patients may have elevated cTn (cardiac troponin) values and marked decreases in ejection fraction due to sepsis caused by endotoxin, with myocardial function recovering completely once the sepsis is treated". Despite their clinical utility, elevated cardiac biomarkers alone do not confirm myocardial injury or ventricular dysfunction. Histopathological evidence remains the definitive proof of myocardial injury, but myocardial tissue is rarely available in clinical practice, making imaging indispensable. Coronary angiography and echocardiography are therefore essential for differentiating the underlying cause, assessing severity, and guiding management." (Page 4, line 25 – Page 5, line 2)
- As a result of the above revisions, the following sentence was removed: "Several reviews on sepsis-related cardiomyopathy suggest that bacterial toxins can directly induce myocardial toxicity at the cardiomyocyte level, leading to cell injury or death. The most recent international definition of myocardial infarction acknowledges that myocardial injury with elevated cTn levels in sepsis, often

accompanied by reversible ventricular dysfunction, may result from endotoxin exposure" (Page 6, lines 1-5).

1) Electrocardiogram is a simple and widely used examination, and electrocardiographic abnormalities are commonly employed to define various cardiomyopathies (doi:10.1161/CIR.0000000000001062). The authors should explain why they chose not to include electrocardiographic abnormalities in their screening criteria

   We thank the reviewer for this suggestion. We agree that electrocardiographic (ECG) abnormalities are commonly used in the characterization of cardiomyopathies and can indicate myocardial involvement in sepsis. We have therefore revised our eligibility criteria to include studies that report predefined ECG abnormalities consistent with acute myocardial injury, even when biomarker or imaging data are unavailable. The following section has been altered accordingly: "Additionally, studies must provide evidence of cardiomyocyte injury, coronary microvascular dysfunction, or ventricular dysfunction, assessed by histopathology, <u>electrocardiography</u>, or imaging techniques such as coronary angiography, echocardiography or MRI including, where available, measurements of coronary flow or myocardial perfusion. (Page 4, lines 4-8).

2) In the Introduction, some referenced studies (e.g., references 10 to 13) were conducted in mice rather than humans. The authors should specify the experimental model used for each affirmation

   We thank the reviewer for this comment. We agree that it is important to distinguish between findings from animal models and those from human studies. In the revised manuscript, we have clarified that the cited studies (references 10–16) were conducted in experimental animal models (Page 3 line 27 – Page 4 line 14). We have not specified the exact type of different animal models in the Introduction, as this level of detail is considered beyond what is proportionate for a protocol introduction, but such information will be extracted and reported where relevant in the final review.

3) Why did the authors limit inclusion to patients over 18 years of age? As they noted, a retrospective analysis does not raise ethical concerns regarding pediatric populations

   We thank the reviewer for this observation. Our decision to limit inclusion to patients aged 18 years and older was based on both clinical and methodological considerations. Sepsis in children and neonates differs fundamentally from adult sepsis in terms of epidemiology, immune response, myocardial physiology, and clinical management. Pediatric studies often use different definitions of sepsis, distinct biomarker thresholds, and age-specific echocardiographic criteria for myocardial function, making direct comparison with adult populations problematic. Because our review aimed to provide a coherent synthesis of myocardial injury and dysfunction in adult sepsis, we restricted inclusion to patients ≥18 years of age. To address this important point we have inserted the following text: "Pediatric populations (<18 years) will be excluded because sepsis in children differs from adults in epidemiology, pathophysiology, biomarker thresholds, and

echocardiographic criteria, making direct comparison inappropriate" (Page 8, lines 13-15).

4) Patients with a known history of cardiomyopathy should be excluded from the analysis to ensure that any abnormal cardiologic findings are specific to sepsis-related myocardial injury and not due to pre-existing cardiac conditions

   We thank the reviewer for this suggestion. We acknowledge that sepsis patients often have a range of pre-existing cardiovascular comorbidities, including cardiomyopathy. However, most studies in this field do not consistently report or stratify findings according to such comorbidities. Excluding these patients or attempting to extract incomplete data would therefore not be feasible and would risk substantially narrowing the available evidence base. For this reason, we did not include pre-existing cardiomyopathy as an exclusion criterion and did not plan to extract these data. We recognize this as a limitation and will highlight it in the Discussion of the scoping review itself.

5) Regarding the search strategy, terms related to cardiac abnormalities are missing. Other studies have reported the use of various imaging techniques in the assessment of sepsis-related myocardial injury (doi:10.1097/CCM.0000000000002851, doi:10.1186/s13054-018-2043-8), including echocardiography, magnetic resonance imaging, left ventricular ejection fraction, Tei index, tricuspid annular plane systolic excursion, tricuspid S' peak systolic velocity, and cardiac output

   We thank the reviewer for this constructive suggestion. We recognize that sepsis-related myocardial injury has been assessed using a variety of imaging modalities and parameters, including echocardiography, cardiac MRI, left ventricular ejection fraction, Tei index, tricuspid annular plane systolic excursion, tricuspid S′ peak systolic velocity, and cardiac output, as reported in the cited studies. In our test searches, however, the inclusion of highly specific imaging terms markedly reduced sensitivity and excluded many potentially relevant studies. Following the advice ofour medical librarian, we therefore structured the search to remain broad, using established MeSH terms (e.g., *Myocardium*, *Cardiomyopathies*, *Troponin*, *Creatine Kinase*, *Natriuretic peptides*, *Cardiac Imaging Techniques*) as well as text word searches for *cardio*, *myocard*, *cardiac*, *contractile dysfunction*, and *ventricular dysfunction*. This strategy was intended to balance sensitivity and specificity, ensuring comprehensive coverage of the literature while minimizing the risk of missing eligible studies. To increase the transparency of our search strategy, the following text has been inserted in the manuscript: "Highly specific imaging terms (e.g. cardiac output, LVEF) were not included in the search string, as test searches showed thattheir use markedly reduced sensitivity and excluded potentially relevant studies. Following the advice of a medical librarian, we therefore applied broader MeSH terms and text words to ensure comprehensive coverage of the literature" (Page 8, lines 26-30).

6) Sepsis can be caused not only by bacteria but also by other microorganisms such as viruses, parasites, and fungi. The authors should justify why their analysis focuses exclusively on bacterial causes.

We thank the reviewer for this important observation. We fully acknowledge that sepsis can arise from a wide range of pathogens, including viruses, parasites, and fungi. However, the present scoping review was deliberately focused on bacterial sepsis, as our research question aimed to examine the direct cardiotoxicity of bacteria and bacterial toxins. This focus reflects both the epidemiology of sepsis, where bacteria remain the most common cause, and the existing mechanistic evidence base, which has historically centered on bacterial pathogens and their toxins (e.g., endotoxin, exotoxins). By restricting our scope to bacterial sepsis, we sought to ensure a manageable and coherent review, while allowing for detailed mapping of the literature on direct bacterial myocardial toxicity. We agree that the role of viral, fungal, and parasitic pathogens in sepsis-related myocardial injury is also important and warrants a separate dedicated review. However, to clarify this important point we have added the following to the manuscript: "While sepsis may result from diverse pathogens, including viruses, parasites, and fungi, these etiologies fall outside the scope of the present review" (Page 6, lines 15-17).

Reviewer: 2

Here, the authors propose their methodology for carrying out a scoping review of the cardiac effects of bacterial toxins (direct effect) during septic shock in humans Although the subject itself is particularly interesting and the inclusion and exclusion criteria and the review methodology are very well described in accordance with rigorous standards and approaches, I fail to understand the point of publishing the protocol for this review. This is a personal opinion that only I can express. Once again, I find the subject fascinating and one that could have a major impact on developing future research projects: targeted and personalised therapy, biomarker research, personalised approach to the host-pathogen relationship, etc. So yes, providing information about such a research project is interesting, but the methodology is less exciting, especially in the age of AI.

We thank the reviewer for this perspective and for highlighting the importance of the topic. We appreciate the point that methodology sections may seem less engaging, particularly in the current era of rapidly evolving tools such as AI. Nevertheless, publishing a protocol provides transparency, allows constructive feedback before the review is undertaken, and helps minimize duplication of work in an area where studies are heterogeneous and definitions vary. Although AI tools are increasingly being developed to assist with parts of the review process, none have yet been validated or endorsed by major guideline-setting organizations or publishing bodies as a replacement for established scoping review methodology. This highlights the continued importance of making protocols publicly available. By sharing our planned methods, we aim to strengthen the credibility and reproducibility of the final review and to provide a useful reference for others working in the field.
